# Testosterone deficiency causes penile fibrosis and organic erectile dysfunction in aging men. Evaluating association among Age, TDS and ED

**DOI:** 10.1186/1471-2482-12-S1-S24

**Published:** 2012-11-15

**Authors:** Fabrizio Iacono, Domenico Prezioso, Antonio Ruffo, Ester Illiano, Leo Romis, G Di Lauro, Giuseppe Romeo, Bruno Amato

**Affiliations:** 1 Department of Urology, University “Federico II” of Naples. Via Pansini, 5 - 80131 – Naples, Italy; 2 Department of General, Geriatric, Oncologic Surgery and Advanced Technologies, University “Federico II” of Naples. Via Pansini, 5 - 80131 – Naples, Italy; 3 'Hospital Santa Maria delle grazie', Via Domitiana località La Schiana – 80078 – Pozzuoli, Naples, Italy

## Abstract

**Introduction:**

We studied the possible correlation between age, testosterone deficiency, cavernosal fibrosis and erectile dysfunction (ED).

**Methods:**

47 patients with ED were enrolled between September 2010 and October 2011. IIEF-EF score, NPTR test using the Rigiscan method, total and free testosterone levels, and cavernosum biopsy were carried out on all patients. Patients aged 65 or over were defined as Old Age (OA) while patients under 65 were defined Young age (YA). The strength of the relationships found was estimated by Odds Ratio.

**Results:**

74% of patients with values of over 52% collagen fibers in the corpora cavernosa were found to have organic ED. A significant difference was found in age, percentage of collagen fibers, testosterone levels between patients with Positive Rigiscan (PR) and Negative Rigiscan (NR). Hypotestosteronaemia increased the risk of ED with PR (OR: 21.4, 95% CI: 20.2-22.6) and in both young age patients (OR: 4.3, 95% CI: 2.4-6.2) and old age patients (OR: 15.5, 95% CI: 13.4-17.6). Moreover cavernosal fibrosis increased the risk of ED with PR in both young age patients (OR: 8.2, 95% CI: 6.4-10.0 and old age patients (OR: 24.6, 95% CI: 20.8-28.4).

**Conclusions:**

This study demonstrates a strong association among age, testosterone deficiency, cavernosal fibrosis and ED with PR. Age, testosterone deficiency and cavernosal fibrosis are potentially correctable factors of cavernosal fibrosis and organic ED. Further, prospective studies are needed to evaluate if testosterone treatment, alone or in association with PDE5 inhibitors, may lower the risk of cavernosal fibrosis or decrease the severity the fibrosis in ED patients.

## Background

Male ageing causes alterations in both libido and erectile function. Although the majority of men over fifty are still interested in sex, the prevalence of sexual activity declines with age: 73% of patients who are 57 to 64 years of age are able to have regular sexual relations (53% of patients who are 65-74 years of age and only 26% of patients who are 75 to 85 years of age) [1]. One of the major causative factors of this is the elevated incidence of Erectile Dysfunction (ED) that occurs in the sixth and seventh decade of life. Erection is a complex phenomenon involving the interaction of the autonomic nervous system, cardiovascular system and local neurotransmitters. Sexual stimulus brings about blood flow into the corpus cavernosum and the consequent penile rigidity is maintained by means of a veno-occlusive mechanism. This is enabled by the particular microarchitecture of the corpus cavernosum which consents a sophisticated haemodynamic mechanism.

Androgens are essential for the development, growth and maturation of the erectile tissues. In the animal model testosterone suppression lead to corpora cavernosum atrophy with concomitant structural alterations of the dorsal nerve of the penis, endothelial alterations, reduction of the smooth muscle component and increase in the deposition of extracellular matrix and cavernosal fibrosis. Androgens, acting on the haemostasis in the corpora cavernosa, regulate the growth of smooth muscle and protein synthesis of the connective tissue of the corpora cavernosa and a decrease in their production could give rise to the switch from elastic fibers to collagen fibers, which is the basis of cavernosal fibrosis [2,3]. In the brain, low testosterone levels are associated with a reduction in erectile signaling. Studies in hypogonadal patients have shown that testosterone replacement results in significant increase in brain activity in response to sexual stimulation, to levels similar to those seen in men with normal testosterone. Recent experimental evidence showed that Testosterone also regulates the expression of phosphodiesterasis type 5 (PDE5) [4]. As age advances the gonadal steroid hormones and, in particular, testosterone production decreases [5], nerve conduction slows and the efficiency of the vascular microcirculation of the penis is reduced.

Furthermore it has been previously shown that as age advances the collagen/elastic fibers ratio in the corpora cavernosa increases [6]. Therefore, it seems that the aging and the testosterone production decrease can lead to a progressive fibrotic process of the corpora cavernosa which is related to testosterone.

However, to date it is still not clear what degree of fibrosis gives rise to erectile dysfunctions in man, nor the role of testosterone in the pathogenesis of cavernosal fibrosis has yet been clarified. This study seeks to evaluate the functional and microstructural alterations that take place in the cavernosa tissue in a group of over fifty ED patients with and without hypotestosteronaemia, in order to establish the correlation and causal relationship among age, testosterone deficiency and cavernosal fibrosis in men affected with ED.

## Materials and methods

The study consisted in a descriptive investigation conduced from September 2010 and October 2011. Inclusion criteria were: over fifty years of age, male patients with stable marital relations and affected by Erectile Dysfunction (ED). Exclusion criteria were: diabetes mellitus and other metabolic disorders (Impaired Glucose Tolerance, Impaired Fasting Glucose, Metabolic Syndrome and congenital or acquired dyslipidemia), obesity, alcoholism, smoking, hypertension, cardiovascular disease, Neurogenic syndrome (Multiple sclerosis, Multiple atrophy, Parkinson's disease, Tumors, Stroke, Disk disease, Spinal cord disorders, Polyneuropathy, Uraemia), Peyronie's disease, Penile fracture, Congenital curvature of penis, Micropenis, Hypospadias, Epispadias, Hyperprolattinemia, Hyper- and Hypothyroidism, Cushing's disease, drug assumption (antihypertensives, antidepressants, antipsychotics, antiandrogens, antihistamines, heroin, cocaine and methadone), radiotherapy (pelvis or retroperitoneum) and lower pelvic surgery (oncological pelvic surgery, lower urinary and genital tract surgery). We enrolled 47 patients presenting at the Andrology Department of our Clinic. At visit patients were evaluated by means of detailed medical and sexual history, clinical examination, laboratory investigations (Total and Free Testosterone), strumental examination (NPTR with Rigiscan) and biopsies of the corpora cavernosa. The study was performed in compliance with the guidelines of our institutional ethics committee.

Written informed consent was obtained from all patients.

Patients aged 65 or over were defined as Old Age (OA) while patients under 65 were defined Young age (YA). We asked all patients to complete the International Index of Erectile Function (IIEF) questionnaire: the IIEF domain were calculated and ED grading was determined: absent of ED (IIEF score 26 to 30), mild ED (IIEF score 17 to 25), moderate ED (EF score 11 to 16) and severe ED (IIEF score < 10) [7].

All patients underwent nocturnal penile tumescence and rigidity test (NPTR) with Rigiscan for three consecutive nights. Normal erectile function was defined with the recording of at least one erection (70 out of 100% tip rigidity lasting for at least 10 min during either night) [8]: we considered Negative Rigiscan (NR) patients who had an erectile event of at least 70% rigidity recorded on the tip of the penis, which lasted for 10 minutes or more, and Positive Rigiscan (PR) remaining patients (Table 1). We practiced this test with the intent of differentiating organic and psychogenic erectile dysfunction [9]. Serum testosterone concentrations were measured using the DPC Coat-A-Count RIA kit, which has an intra- and interassay coefficient of variation (CV) of < 10% while sex hormone-binding globulin (SHBG) were measured by ELISA technique (DRG Diagnostics, Marburg, Germany). We calculated the measurement of free testosterone (FT) from measured total testosterone (tT) and SHBG. These measurement were obtained between 07.00 am and 11.00 am. There are no accepted lower limits of normal and it is unclear whether geographically different threshold depend on ethnic differences or on the physician's perception. However there is general agreement that total testosterone levels above 12 nmol/L (346 ng/dL) or free testosterone levels above 250 pmol/L (72 pg/mL) do not require testosterone substitution. Similarly, testosterone supplementation should be started according to the reference levels given in the recommendations of the ISA-ISSAM-EAU [10] when serum total testosterone levels are below 8 nmol/L (231 ng/dL) or free testosterone levels are below 180 pmol/L (52 pg/mL) and when serum total testosterone levels are between 12 and 8 nmol/L or free testosterone levels are between 250 and 180 pmol/L in patients with symptoms of testosterone deficiency. So we considered Normal Testosterone (NT) patients who had total and testosterone levels above 12 nmol/L (346 ng/dL) and free testosterone levels above 250 pmol/L (72 pg/mL), and Low Testosterone (LT) patients who had testosterone levels below 12 nmol/L (346 ng/dL) and free testosterone levels below 250 pmol/L (72 pg/mL). Biopsies of the corpora cavernosa were performed in all patients, by means of an automatic needle, under local anesthesia induced by 1cc of 5% carbocaine and an incision of 0.5 cm at a distance of 1 cm from the balano-preputial groove [11]. Connective tissue specimens were fixed in neutral buffered 10% formalin for 12-24 hours, washed in ethanol, processed by standard methods, embedded in paraffin, sectioned at 4µm and stained with haematoxylin and eosin, and with Masson's Trichomome method by autostainer. The connective tissue presents in specimens stained with Masson's Trichome method was evaluated by computer image analysis (Eureka Interface System - Menarini Diagnostic) with an analysis per area.

The study population was divided into two groups: patients diagnosed with PR and patients NR, and compared for mean age, percent collagen fibers content, total and free testosterone levels by Student t test (p < 0.005). Moreover, further analysis were carried out in both group YA and OA to quantify associations among hypotestosteronaemia, cavernosal fibrosis and PR (95% CI) in order to establish whether cavernosal fibrosis and/or hypotestosteronaemia represent epiphenomena of old age or the are factors involved in the onset of organic ED.

**Table 1:** Patients' data

***Age***	***OA***	***IIEF-EF***	***Rigiscan***	***Rigiscan***	***Rigiscan (+)***	***T tot***	***T free***	***LT***	***Biopsy***
*Years old*	*N* (*%*)	*Average score*	*Tip*	*Base*	*N°* (*%*)	*ng/dl*	*ng/dl*	*N* (*%*)	*% collagen fibers*
***66+/-3***	**26 (55)**	**13.5 +/- 4.3**	**50.4+/-2.5**	**50.5+/-2.7**	**35 (75)**	**443+/-22**	**6.1+/-0.3**	**20 (43)**	**53.5+/-2.7**

Mean results of all the patients and all parameters investigated. 47 patients: Age, number (and %) of patients over 65, IIEF-EF score, Rigiscan test, number (and %) of patients with PR, total and free testosterone, number (and %) of patients with hypotestosteronaemia, collagen fibers content *per area* present in corpora cavernosa.

## Results and discussion

**Figure 1:**
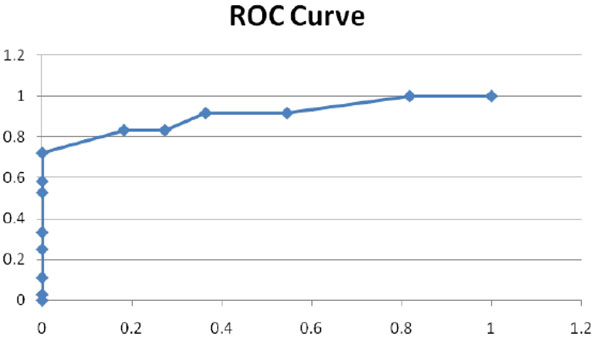
**ROC Curve.** The area under the ROC curve is 0.91 (p<0.001). Confidence interval is 0.824 – 0.989.

Forty-seven men, mean age 66 (+/- 3), were enrolled. IIEF score, penile rigidometry, patients with PR, total and free testosterone levels; number of patients diagnosed with hypotestosteronaemia and percent of collagen fibers cavernosal are reported (Table 1). When the whole group was divided according to a positive or negative Rigiscan a highly significant difference was found (p < 0.001) (Fig.1) between age, degree of cavernosal fibrosis, and total and free testosterone levels. As we can see in the group of patients with PR there is the presence of a higher percentage of collagen fibers, with a concomitant decrease of smooth muscle cells. Furthermore there is a remarkable difference of the serum free and total testosterone levels between the PR (364 +/- 18 tT and 5.5 +/- 0.4 FT) and NR groups (671 +/- 24 and 7.8 +/- 0.5) (Table 2).

**Table 2:** Student T test results on mean ages, percentage of collagen fibres, free and total testosterone levels in both the PR and NR. Results' Student T in both populations of patients: organic ED and psychogenic ED.

	***Positive Rigiscan***	***Negative Rigiscan***	***P***
Age	70	56	.000 *
% Collagen Fibers	57.1	47.8	.000*
Total Testosterone	364 +/-18	671 +/-24	.000*
Free Testosterone	5.5 +/-0.4	7.8 +/-0.5	.000*

* significant difference between the means.

A 2x2 chart was drawn up, taking the percent collagen fibers content per area as an independent variable and seeking to establish the most accurate fibrosis value (cut off) to identify patients who had PR. This value was fixed at 52%, that was reputed the best compromise between higher sensitivity and specificity. So the subjects under study were subdivided into two groups according to the collagen fibers content present in the corpora cavernosa: the first group, in which the collagen fibers at biopsy were less than 52%, was denominated Low Fibrosis (LF), and the second group, in which the collagen fibers at biopsy were above 52%, was denominated High Fibrosis (HF).

The strength of the relationships found among the data was estimated by Odds Ratio, with 95% confidence intervals (95% CI). Selecting a 52% cut off value of percentage of collagen fibers for cavernosal fibrosis we obtained 74% sensitivity and 100% specificity to identify patients who had NPTR test positive suggesting a diagnosis of organic ED. Thus a fibrosis > 52% is associated with PR in 74% of cases (Table 3) whereas below this value PR patients were not found. The strength of the relationships found between hypotestosteronaemia/cavernosal fibrosis and hypotestosteronaemia/Rigiscan's outcomes was estimated by Odds Ratio (Table 4).

**Table 3:** Percent of collagen fibres in corpora cavernosa in patients with PR and NR. Study group divided according to the percentage collagen fibres revealed at corpora cavernosa biopsy (a value >52% is considered positive for high fibrosis) and Rigiscan’s results.

	Positive Rigiscan	Negative Rigiscan	Total
HF	26 (55%)	0 (0%)	26 (55%)
LF	9 (20%)	12 (25%)	21 (45%)
Total	35 (75%)	12 (25%)	47(100%)

**Table 4:** Results' OR. This table reports OR and CI’s results for LT to HF, LT to PR, OA to LT, OA to HF, OA to PR, 95% confidence limits and intervals.

***Variables***	***OR***	***C.I. (95%)***
***LT to HF***	**13.1**	**11.3-14.9**
***LT to PR***	**21.4**	**20.2-22.6**
***OA to LT***	**3.4**	**2.5-4.3**
***OA to HF***	**2.6**	**1.5-3.7**
***OA to PR***	**7.5**	**5.1-9.9**

Androgens play a crucial role in the maturation and differentiation of erectile tissue. However, their role in the erectile function of man is still on debate. In fact, in the animal model, it has been shown that hypotestosteronaemia is responsible for corpora cavernosa atrophy, nervous and endothelial structures modifications, smooth muscle component decrease, collagen fibers neo-deposition, subtunical adipocyte deposits and enzymatic inhibition of nitric oxide synthases [5], while testosterone could play a role in erectile signaling in the brain and on the other hand could regulate the expression of PDE5 [3]. However, androgen deficiency is "quite common" in men presenting with ED, particularly if it is associated with uncontrolled diabetes, high total cholesterol and anaemia [12]. This study demonstrates that amongst patients affected with mild to severe ED, the cavernosal fibrosis is greater in the organic form than in the psychogenic form although we know that NPTR has many advantages in differentiating psychogenic from organic ED, however, several questions related to its physiological mechanisms do exist [13]. The percentage of collagen fibers lower than 52% excludes an organic component in the etiopathogenesis of ED, whereas a content greater than 52% per area is associated to a 74% of sensibility in the diagnosis of ED with Positive Rigiscan. In patients who have undergone to radical prostatectomy, the process of collagenization of the corpora cavernosa takes place which could be associated with post-prostatectomy ED [14]. However, it is clear that many other etiologies such as vascular damage, smoking and metabolic disorders can give rise to organic ED due to an increase in cavernosal fibrosis [15]. To date the evaluation of cavernosal fibrosis has only been carried out by qualitative methodology which does not estimate the degree of fibrosis which is associated to organic erectile dysfunction. We have also demonstrated that the degree of fibrosis is directly correlated with age and inversely correlated with total and free testosterone levels. The increase of cavernosal fibrosis with age has been previously reported by other authors [16]. Furthermore the present study confirms the association between age and cavernosal fibrosis by means of the OR. In our opinion, the fact that fibrosis might be a consequence of Hypotestosteronaemia, commonly found in advanced age, is of great interest. An outcome very interesting is that hypotestosteronaemia, cavernosal fibrosis and organic erectile dysfunction were rare in younger patients but the presence of cavernosal fibrosis and/or hypotestosteronaemia considerably increased the risk of organic DE. This seems to confirm the strong association among low testosterone levels, cavernosal fibrosis and erectile dysfunction.

## Conclusions

The aim of this study is to establish a connection between the decrease of free and total testosterone levels and the progressive cavernosal fibrosis, that plays a key role in the etiology of ED. Androgens deficiency in the aging male has become a topic of great interest and debate due the increasing percentage of the population belonging to the older age group. The data shows that serum testosterone levels fall gradually with the age (TDS) [17] with a concomitant increase of ED [18]. In our study we have found that in the population of patients affected by ED with a positive Rigiscan there are low testosterone/free testosterone levels (364 ng/dL and 5.5 ng/dL) comparing to the group of patients with negative Rigiscan and normal testosterone/free testosterone levels (Table 2). The correlation among androgens decrease, cavernosal fibrosis and ED seems evident. Therefore the ED associated to TDS (LOH) could be linked to the testosterone decrease that characterizes this syndrome and to the consequent cavernosal fibrosis, final step toward the ED. The use of PDE5i have been used to inhibit the cavernosal fibrosis resulting in patients underwent radical prostatectomy [19,20]. Therefore the association of Testosterone replacement therapy and PDE5i could be effective to contrast the cavernosal fibrosis linked to the aging and to the testosterone decrease. The same effect on the corpora cavernosa could be obtained with the assumption of natural compounds containing Protodioscin and Phlorotannins with antioxidant and anti-fibrotic action [21,22].

## List of abbreviations used

CI: Confidence Intervals; CV: Coefficient of Variation; ED: Erectile Dysfunction; HF: High Fibrosis; IIEF: International Index of Erectile Function; LF: Low Fibrosis; LT: Low Testoserone; FT: Free Testosterone; tT: Total Testosterone; NPTR: Nocturnal Penile Tumescence and Rigidity; NR: Negative Rigiscan; NT: Normal Testosterone; OR: Old Age; OD: Odds Ratio; PDE5: Phosphodiesterasis type 5; PR: Positive Rigiscan; SHBG: Sex Hormone Binding Globulin; YA: Young Age

## Competing interests

The authors declare that they have no competing interests.

## Authors' contributions

FI: conception and design, interpretation of data, given final approval of the version to be published; DP, AR, EI, GR, LR, GdL: acquisition of data, drafting the manuscript, given final approval of the version to be published; BA: critical revision, interpretation of data, given final approval of the version to be published.
